# Bis{[2-(dimethyl­amino)­phen­yl](trimethyl­sil­yl)meth­yl}cadmium

**DOI:** 10.1107/S1600536812001079

**Published:** 2012-01-18

**Authors:** Min Li

**Affiliations:** a Department of Biochemistry, Changzhi University, Shanxi, People’s Republic of China

## Abstract

In the crystal structure of the title compound, [Cd(C_12_H_20_NSi)_2_], the Cd^II^ cation is coordinated by two N and two C atoms within an irregular polyhedron. The four Cd—*X* (*X* = C, N) bond lengths are in the range 2.166 (4)–2.513 (4) Å.

## Related literature

For structures of related Cd–alkyl and Cd–aryl complexes, see: Schmidbaur *et al.* (1981 ▶). For the synthesis of related compounds, see: Tong *et al.* (2011 ▶).
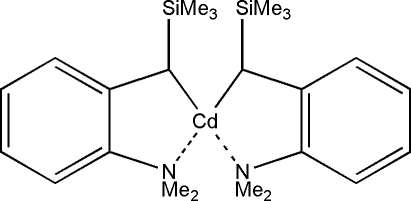


## Experimental

### 

#### Crystal data

[Cd(C_12_H_20_NSi)_2_]
*M* *_r_* = 525.16
Triclinic, 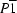

*a* = 9.341 (6) Å
*b* = 11.011 (5) Å
*c* = 13.605 (8) Å
α = 87.80 (4)°
β = 88.31 (3)°
γ = 77.30 (5)°
*V* = 1363.7 (13) Å^3^
*Z* = 2
Mo *K*α radiation
μ = 0.90 mm^−1^
*T* = 230 K
0.40 × 0.30 × 0.30 mm

#### Data collection

Bruker SMART APEX CCD diffractometer
Absorption correction: multi-scan (*SADABS*; Sheldrick, 1996 ▶) *T*_min_ = 0.715, *T*_max_ = 0.774
5641 measured reflections
4701 independent reflections
4067 reflections with *I* > 2σ(*I*)
*R*_int_ = 0.015

#### Refinement

*R*[*F* ^2^ > 2σ(*F* ^2^)] = 0.041
*wR*(*F* ^2^) = 0.086
*S* = 1.09
4701 reflections
280 parameters
H atoms treated by a mixture of independent and constrained refinement
Δρ_max_ = 0.60 e Å^−3^
Δρ_min_ = −0.24 e Å^−3^

### 

Data collection: *SMART* (Siemens, 1996 ▶); cell refinement: *SAINT* (Siemens, 1996 ▶); data reduction: *SAINT*; program(s) used to solve structure: *SHELXS97* (Sheldrick, 2008 ▶); program(s) used to refine structure: *SHELXL97* (Sheldrick, 2008 ▶); molecular graphics: *SHELXTL* (Sheldrick, 2008 ▶); software used to prepare material for publication: *SHELXL97*.

## Supplementary Material

Crystal structure: contains datablock(s) I, global. DOI: 10.1107/S1600536812001079/nc2262sup1.cif

Structure factors: contains datablock(s) I. DOI: 10.1107/S1600536812001079/nc2262Isup2.hkl

Additional supplementary materials:  crystallographic information; 3D view; checkCIF report

## Figures and Tables

**Fig. 1.**
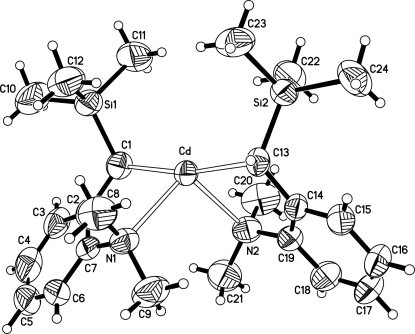
Crystal structure of the title compound with labelling and displacement ellipsoids drawn at the 50% probability level.

## supplementary crystallographic information

### Comment

Although several Cd complexes were reported Cd-alkyl or Cd-aryl complexes are still rare (Schmidbaur, *et al.*, 1981). For the synthesis of such complexes the corresponding lithium compounds are useful reactands (Tong, *et al.*, 2011). In the course of synthesizing new Cd complexes we obtained crystals of the title compound which were characterized by single crystal X-ray diffraction.

In the crystal structure of the title compound the Cd(II) cation are 4-fold coordinated by two nitrogen and two carbon atoms. The Cd–C bond lengths amount to 2.166 (4)(Cd—C1) and 2.169 (3)(Cd—C13)Å, and the Cd–N bond lengths are 2.503 (3) Å (Cd—N2) and 2.513 (4) Å(Cd—N1). The C1- Cd1—C13 bond angle is 167.3 (1)°) and the coordination environment around the Cd cations can be described as a irregular polyhedron.fragment.

### Experimental

All manipulations were carried out under argon using standard Schlenk techniques. A solution of 2-dimethylamino-*R*-trimethylsilylbenzyl-Li (0.43 g, 0.2 mmol in 20 ml hexane) was slowly added to a stirred suspension of CdCl_2_ (0.13 g, 0.1 mmol) in hexane (*ca* 10 ml) at *ca* 273 K. The resulting mixture was slowly warmed to room temperature and stirred for an additional 5 h to give a white precipitate of LiCl. The mixture was filtered and the filtrate was carefully concentrated in vacuum to give colorless crystal of the title compound.

### Refinement

The H atoms were positioned with idealized geometry and refined isotropic with *U*_iso_(H) = 1.2 *U*_eq_(C) (1.5 for methyl H atoms).

### Crystal data

**Table:**

[Cd(C_12_H_20_NSi)_2_]	*Z* = 2
*M**_r_* = 525.16	*F*(000) = 548
Triclinic, *P*1	*D*_x_ = 1.279 Mg m^−^^3^
*a* = 9.341 (6) Å	Mo *K*α radiation, λ = 0.71073 Å
*b* = 11.011 (5) Å	Cell parameters from 3359 reflections
*c* = 13.605 (8) Å	θ = 2.3–27.7°
α = 87.80 (4)°	µ = 0.90 mm^−^^1^
β = 88.31 (3)°	*T* = 230 K
γ = 77.30 (5)°	Stick, colorless
*V* = 1363.7 (13) Å^3^	0.40 × 0.30 × 0.30 mm

### Data collection

**Table:**

Bruker SMART APEX CCD diffractometer	4701 independent reflections
Radiation source: fine-focus sealed tube	4067 reflections with *I* > 2σ(*I*)
graphite	*R*_int_ = 0.015
phi and ω scans	θ_max_ = 25.0°, θ_min_ = 1.5°
Absorption correction: multi-scan (*SADABS*; Sheldrick, 1996)	*h* = −10→11
*T*_min_ = 0.715, *T*_max_ = 0.774	*k* = −12→13
5641 measured reflections	*l* = −16→12

### Refinement

**Table:**

Refinement on *F*^2^	Primary atom site location: structure-invariant direct methods
Least-squares matrix: full	Secondary atom site location: difference Fourier map
*R*[*F*^2^ > 2σ(*F*^2^)] = 0.041	Hydrogen site location: inferred from neighbouring sites
*wR*(*F*^2^) = 0.086	H atoms treated by a mixture of independent and constrained refinement
*S* = 1.09	*w* = 1/[σ^2^(*F*_o_^2^) + (0.0331*P*)^2^ + 0.7748*P*] where *P* = (*F*_o_^2^ + 2*F*_c_^2^)/3
4701 reflections	(Δ/σ)_max_ = 0.001
280 parameters	Δρ_max_ = 0.60 e Å^−^^3^
0 restraints	Δρ_min_ = −0.24 e Å^−^^3^

### Special details

**Table:**

Geometry. All e.s.d.'s (except the e.s.d. in the dihedral angle between two l.s. planes) are estimated using the full covariance matrix. The cell e.s.d.'s are taken into account individually in the estimation of e.s.d.'s in distances, angles and torsion angles; correlations between e.s.d.'s in cell parameters are only used when they are defined by crystal symmetry. An approximate (isotropic) treatment of cell e.s.d.'s is used for estimating e.s.d.'s involving l.s. planes.
Refinement. Refinement of *F*^2^ against ALL reflections. The weighted *R*-factor *wR* and goodness of fit *S* are based on *F*^2^, conventional *R*-factors *R* are based on *F*, with *F* set to zero for negative *F*^2^. The threshold expression of *F*^2^ > σ(*F*^2^) is used only for calculating *R*-factors(gt) *etc*. and is not relevant to the choice of reflections for refinement. *R*-factors based on *F*^2^ are statistically about twice as large as those based on *F*, and *R*- factors based on ALL data will be even larger.

### Fractional atomic coordinates and isotropic or equivalent isotropic displacement parameters (Å^2^)

**Table:**

	*x*	*y*	*z*	*U*_iso_*/*U*_eq_	
Cd	0.25382 (3)	0.99621 (2)	0.753955 (19)	0.04535 (11)	
Si2	0.52432 (12)	0.76494 (10)	0.83055 (8)	0.0538 (3)	
Si1	0.39659 (12)	1.23578 (11)	0.67425 (9)	0.0577 (3)	
C13	0.3230 (4)	0.8293 (3)	0.8458 (3)	0.0447 (9)	
N1	0.0380 (3)	1.1417 (3)	0.8311 (2)	0.0513 (8)	
C14	0.2231 (4)	0.7414 (3)	0.8309 (3)	0.0455 (8)	
C2	0.0830 (4)	1.2435 (3)	0.6727 (3)	0.0498 (9)	
C3	0.0275 (5)	1.3305 (4)	0.5981 (3)	0.0672 (12)	
H3	0.0867	1.3392	0.5433	0.081*	
N2	0.1275 (3)	0.8475 (3)	0.6757 (2)	0.0566 (8)	
C7	−0.0104 (4)	1.2342 (3)	0.7535 (3)	0.0506 (9)	
C1	0.2359 (4)	1.1633 (4)	0.6616 (3)	0.0504 (9)	
C16	0.1279 (5)	0.5640 (4)	0.8942 (4)	0.0749 (13)	
H16	0.1255	0.5036	0.9436	0.090*	
C15	0.2164 (4)	0.6483 (4)	0.9027 (3)	0.0589 (10)	
H15	0.2735	0.6429	0.9581	0.071*	
C19	0.1321 (4)	0.7478 (3)	0.7502 (3)	0.0523 (9)	
C24	0.5904 (5)	0.6124 (4)	0.8995 (4)	0.0893 (16)	
H24A	0.5656	0.6225	0.9681	0.134*	
H24B	0.6950	0.5864	0.8913	0.134*	
H24C	0.5445	0.5506	0.8742	0.134*	
C6	−0.1501 (4)	1.3103 (4)	0.7582 (4)	0.0709 (13)	
H6	−0.2100	1.3035	0.8130	0.085*	
C21	−0.0221 (5)	0.9041 (5)	0.6421 (4)	0.0861 (15)	
H21A	−0.0531	0.8494	0.5980	0.129*	
H21B	−0.0220	0.9825	0.6087	0.129*	
H21C	−0.0884	0.9173	0.6979	0.129*	
C4	−0.1137 (6)	1.4044 (4)	0.6036 (4)	0.0870 (17)	
H4	−0.1484	1.4603	0.5522	0.104*	
C17	0.0436 (6)	0.5697 (4)	0.8127 (4)	0.0874 (16)	
H17	−0.0150	0.5124	0.8057	0.105*	
C18	0.0464 (5)	0.6608 (4)	0.7413 (4)	0.0758 (13)	
H18	−0.0103	0.6643	0.6858	0.091*	
C20	0.2220 (6)	0.8062 (5)	0.5898 (3)	0.0886 (15)	
H20A	0.3199	0.7706	0.6109	0.133*	
H20B	0.2234	0.8761	0.5457	0.133*	
H20C	0.1846	0.7447	0.5566	0.133*	
C8	0.1033 (5)	1.1934 (4)	0.9126 (3)	0.0740 (13)	
H8A	0.1778	1.2344	0.8870	0.111*	
H8B	0.1462	1.1272	0.9578	0.111*	
H8C	0.0285	1.2522	0.9462	0.111*	
C12	0.4331 (5)	1.2721 (5)	0.8026 (3)	0.0826 (14)	
H12A	0.3529	1.3348	0.8268	0.124*	
H12B	0.5221	1.3020	0.8034	0.124*	
H12C	0.4430	1.1981	0.8438	0.124*	
C22	0.5889 (5)	0.7379 (4)	0.6999 (3)	0.0772 (13)	
H22A	0.5444	0.6760	0.6733	0.116*	
H22B	0.6937	0.7096	0.6978	0.116*	
H22C	0.5616	0.8143	0.6618	0.116*	
C10	0.3806 (6)	1.3851 (5)	0.5994 (4)	0.104 (2)	
H10A	0.3671	1.3700	0.5316	0.157*	
H10B	0.4686	1.4156	0.6053	0.157*	
H10C	0.2981	1.4458	0.6231	0.157*	
C9	−0.0774 (5)	1.0803 (5)	0.8704 (3)	0.0813 (14)	
H9A	−0.1421	1.1354	0.9135	0.122*	
H9B	−0.0333	1.0054	0.9063	0.122*	
H9C	−0.1321	1.0602	0.8170	0.122*	
C23	0.6216 (5)	0.8796 (5)	0.8807 (4)	0.0860 (15)	
H23A	0.5968	0.9567	0.8432	0.129*	
H23B	0.7256	0.8472	0.8764	0.129*	
H23C	0.5922	0.8937	0.9483	0.129*	
C5	−0.2017 (6)	1.3948 (4)	0.6845 (5)	0.0915 (18)	
H5	−0.2954	1.4452	0.6892	0.110*	
C11	0.5620 (5)	1.1225 (5)	0.6282 (4)	0.0858 (15)	
H11A	0.5848	1.0524	0.6736	0.129*	
H11B	0.6437	1.1623	0.6224	0.129*	
H11C	0.5424	1.0946	0.5650	0.129*	
H13	0.321 (4)	0.850 (3)	0.912 (3)	0.054 (11)*	
H1	0.248 (4)	1.144 (4)	0.599 (3)	0.064 (13)*	

### Atomic displacement parameters (Å^2^)

**Table:**

	*U*^11^	*U*^22^	*U*^33^	*U*^12^	*U*^13^	*U*^23^
Cd	0.04401 (16)	0.04002 (16)	0.05193 (18)	−0.01013 (11)	−0.00366 (11)	0.00682 (11)
Si2	0.0471 (6)	0.0523 (6)	0.0617 (7)	−0.0106 (5)	−0.0102 (5)	0.0088 (5)
Si1	0.0515 (7)	0.0571 (7)	0.0658 (7)	−0.0177 (5)	0.0040 (5)	0.0123 (5)
C13	0.053 (2)	0.0370 (19)	0.044 (2)	−0.0096 (16)	−0.0077 (17)	0.0026 (16)
N1	0.0472 (18)	0.057 (2)	0.0527 (19)	−0.0189 (15)	0.0022 (14)	0.0037 (15)
C14	0.049 (2)	0.0351 (19)	0.053 (2)	−0.0110 (16)	0.0063 (16)	−0.0040 (16)
C2	0.060 (2)	0.040 (2)	0.053 (2)	−0.0172 (18)	−0.0162 (18)	0.0036 (16)
C3	0.079 (3)	0.052 (2)	0.075 (3)	−0.026 (2)	−0.027 (2)	0.017 (2)
N2	0.053 (2)	0.065 (2)	0.054 (2)	−0.0168 (16)	−0.0082 (15)	−0.0029 (16)
C7	0.039 (2)	0.041 (2)	0.074 (3)	−0.0146 (16)	−0.0096 (18)	−0.0035 (18)
C1	0.058 (2)	0.051 (2)	0.043 (2)	−0.0147 (18)	0.0015 (18)	0.0098 (18)
C16	0.074 (3)	0.048 (3)	0.104 (4)	−0.021 (2)	0.015 (3)	0.011 (2)
C15	0.058 (3)	0.049 (2)	0.068 (3)	−0.0087 (19)	0.0056 (19)	0.0021 (19)
C19	0.045 (2)	0.046 (2)	0.068 (3)	−0.0129 (17)	−0.0020 (18)	−0.0080 (18)
C24	0.070 (3)	0.081 (3)	0.104 (4)	0.006 (3)	−0.008 (3)	0.034 (3)
C6	0.045 (2)	0.050 (3)	0.118 (4)	−0.008 (2)	−0.006 (2)	−0.010 (2)
C21	0.071 (3)	0.091 (4)	0.096 (4)	−0.016 (3)	−0.031 (3)	0.011 (3)
C4	0.092 (4)	0.051 (3)	0.119 (4)	−0.014 (3)	−0.063 (3)	0.022 (3)
C17	0.072 (3)	0.057 (3)	0.142 (5)	−0.035 (3)	0.004 (3)	−0.003 (3)
C18	0.065 (3)	0.066 (3)	0.105 (4)	−0.028 (2)	−0.015 (3)	−0.016 (3)
C20	0.088 (4)	0.122 (4)	0.062 (3)	−0.035 (3)	0.004 (3)	−0.018 (3)
C8	0.070 (3)	0.094 (3)	0.061 (3)	−0.024 (3)	0.000 (2)	−0.012 (2)
C12	0.067 (3)	0.096 (4)	0.093 (4)	−0.035 (3)	0.002 (3)	−0.009 (3)
C22	0.068 (3)	0.078 (3)	0.080 (3)	−0.004 (2)	0.001 (2)	0.006 (2)
C10	0.093 (4)	0.087 (4)	0.142 (5)	−0.045 (3)	−0.022 (3)	0.051 (4)
C9	0.073 (3)	0.090 (4)	0.087 (3)	−0.036 (3)	0.012 (2)	0.017 (3)
C23	0.065 (3)	0.099 (4)	0.102 (4)	−0.032 (3)	−0.024 (3)	0.003 (3)
C5	0.056 (3)	0.050 (3)	0.165 (6)	−0.001 (2)	−0.036 (3)	0.000 (3)
C11	0.062 (3)	0.098 (4)	0.096 (4)	−0.019 (3)	0.028 (2)	−0.001 (3)

### Geometric parameters (Å, °)

**Table:**

Cd—C1	2.166 (4)	C24—H24C	0.9600
Cd—C13	2.169 (3)	C6—C5	1.365 (7)
Cd—N2	2.503 (3)	C6—H6	0.9300
Cd—N1	2.513 (4)	C21—H21A	0.9600
Si2—C13	1.866 (4)	C21—H21B	0.9600
Si2—C23	1.868 (5)	C21—H21C	0.9600
Si2—C22	1.873 (5)	C4—C5	1.369 (8)
Si2—C24	1.881 (4)	C4—H4	0.9300
Si1—C1	1.864 (4)	C17—C18	1.373 (6)
Si1—C12	1.864 (5)	C17—H17	0.9300
Si1—C11	1.868 (5)	C18—H18	0.9300
Si1—C10	1.880 (5)	C20—H20A	0.9600
C13—C14	1.508 (5)	C20—H20B	0.9600
C13—H13	0.94 (4)	C20—H20C	0.9600
N1—C7	1.449 (5)	C8—H8A	0.9600
N1—C9	1.471 (5)	C8—H8B	0.9600
N1—C8	1.472 (5)	C8—H8C	0.9600
C14—C15	1.399 (5)	C12—H12A	0.9600
C14—C19	1.399 (5)	C12—H12B	0.9600
C2—C7	1.397 (5)	C12—H12C	0.9600
C2—C3	1.398 (5)	C22—H22A	0.9600
C2—C1	1.511 (6)	C22—H22B	0.9600
C3—C4	1.391 (7)	C22—H22C	0.9600
C3—H3	0.9300	C10—H10A	0.9600
N2—C19	1.460 (5)	C10—H10B	0.9600
N2—C20	1.468 (5)	C10—H10C	0.9600
N2—C21	1.477 (5)	C9—H9A	0.9600
C7—C6	1.388 (5)	C9—H9B	0.9600
C1—H1	0.89 (4)	C9—H9C	0.9600
C16—C17	1.371 (7)	C23—H23A	0.9600
C16—C15	1.382 (6)	C23—H23B	0.9600
C16—H16	0.9300	C23—H23C	0.9600
C15—H15	0.9300	C5—H5	0.9300
C19—C18	1.387 (5)	C11—H11A	0.9600
C24—H24A	0.9600	C11—H11B	0.9600
C24—H24B	0.9600	C11—H11C	0.9600
			
C1—Cd—C13	167.32 (14)	C5—C6—C7	121.7 (5)
C1—Cd—N2	109.85 (14)	C5—C6—H6	119.2
C13—Cd—N2	78.38 (13)	C7—C6—H6	119.2
C1—Cd—N1	77.71 (14)	N2—C21—H21A	109.5
C13—Cd—N1	110.90 (13)	N2—C21—H21B	109.5
N2—Cd—N1	100.60 (11)	H21A—C21—H21B	109.5
C13—Si2—C23	107.6 (2)	N2—C21—H21C	109.5
C13—Si2—C22	114.40 (19)	H21A—C21—H21C	109.5
C23—Si2—C22	107.7 (2)	H21B—C21—H21C	109.5
C13—Si2—C24	113.6 (2)	C5—C4—C3	120.0 (4)
C23—Si2—C24	107.3 (2)	C5—C4—H4	120.0
C22—Si2—C24	106.0 (2)	C3—C4—H4	120.0
C1—Si1—C12	114.8 (2)	C16—C17—C18	119.4 (4)
C1—Si1—C11	107.2 (2)	C16—C17—H17	120.3
C12—Si1—C11	107.4 (2)	C18—C17—H17	120.3
C1—Si1—C10	112.9 (2)	C17—C18—C19	121.4 (4)
C12—Si1—C10	106.3 (3)	C17—C18—H18	119.3
C11—Si1—C10	107.9 (3)	C19—C18—H18	119.3
C14—C13—Si2	116.9 (3)	N2—C20—H20A	109.5
C14—C13—Cd	109.5 (2)	N2—C20—H20B	109.5
Si2—C13—Cd	110.90 (18)	H20A—C20—H20B	109.5
C14—C13—H13	110 (2)	N2—C20—H20C	109.5
Si2—C13—H13	99 (2)	H20A—C20—H20C	109.5
Cd—C13—H13	110 (2)	H20B—C20—H20C	109.5
C7—N1—C9	113.9 (3)	N1—C8—H8A	109.5
C7—N1—C8	112.1 (3)	N1—C8—H8B	109.5
C9—N1—C8	109.4 (3)	H8A—C8—H8B	109.5
C7—N1—Cd	103.5 (2)	N1—C8—H8C	109.5
C9—N1—Cd	114.1 (3)	H8A—C8—H8C	109.5
C8—N1—Cd	103.3 (2)	H8B—C8—H8C	109.5
C15—C14—C19	116.6 (3)	Si1—C12—H12A	109.5
C15—C14—C13	119.0 (3)	Si1—C12—H12B	109.5
C19—C14—C13	124.3 (3)	H12A—C12—H12B	109.5
C7—C2—C3	116.7 (4)	Si1—C12—H12C	109.5
C7—C2—C1	123.7 (3)	H12A—C12—H12C	109.5
C3—C2—C1	119.6 (4)	H12B—C12—H12C	109.5
C4—C3—C2	121.9 (5)	Si2—C22—H22A	109.5
C4—C3—H3	119.1	Si2—C22—H22B	109.5
C2—C3—H3	119.1	H22A—C22—H22B	109.5
C19—N2—C20	112.1 (4)	Si2—C22—H22C	109.5
C19—N2—C21	113.6 (3)	H22A—C22—H22C	109.5
C20—N2—C21	109.0 (3)	H22B—C22—H22C	109.5
C19—N2—Cd	104.0 (2)	Si1—C10—H10A	109.5
C20—N2—Cd	103.4 (3)	Si1—C10—H10B	109.5
C21—N2—Cd	114.2 (3)	H10A—C10—H10B	109.5
C6—C7—C2	120.5 (4)	Si1—C10—H10C	109.5
C6—C7—N1	120.1 (4)	H10A—C10—H10C	109.5
C2—C7—N1	119.5 (3)	H10B—C10—H10C	109.5
C2—C1—Si1	118.9 (3)	N1—C9—H9A	109.5
C2—C1—Cd	109.3 (2)	N1—C9—H9B	109.5
Si1—C1—Cd	111.72 (19)	H9A—C9—H9B	109.5
C2—C1—H1	107 (3)	N1—C9—H9C	109.5
Si1—C1—H1	99 (3)	H9A—C9—H9C	109.5
Cd—C1—H1	110 (3)	H9B—C9—H9C	109.5
C17—C16—C15	119.7 (4)	Si2—C23—H23A	109.5
C17—C16—H16	120.2	Si2—C23—H23B	109.5
C15—C16—H16	120.2	H23A—C23—H23B	109.5
C16—C15—C14	122.4 (4)	Si2—C23—H23C	109.5
C16—C15—H15	118.8	H23A—C23—H23C	109.5
C14—C15—H15	118.8	H23B—C23—H23C	109.5
C18—C19—C14	120.4 (4)	C6—C5—C4	119.2 (5)
C18—C19—N2	120.3 (4)	C6—C5—H5	120.4
C14—C19—N2	119.3 (3)	C4—C5—H5	120.4
Si2—C24—H24A	109.5	Si1—C11—H11A	109.5
Si2—C24—H24B	109.5	Si1—C11—H11B	109.5
H24A—C24—H24B	109.5	H11A—C11—H11B	109.5
Si2—C24—H24C	109.5	Si1—C11—H11C	109.5
H24A—C24—H24C	109.5	H11A—C11—H11C	109.5
H24B—C24—H24C	109.5	H11B—C11—H11C	109.5
